# Optimising Instrumented Mouthguard Data Analysis: Video Synchronisation Using a Cross-correlation Approach

**DOI:** 10.1007/s10439-025-03679-1

**Published:** 2025-01-21

**Authors:** James Tooby, Steve Rowson, Kevin Till, David Allan, Melanie Dawn Bussey, Dario Cazzola, Éanna Falvey, Kenzie Friesen, Andrew J. Gardner, Cameron Owen, Gregory Roe, Thomas Sawczuk, Lindsay Starling, Keith Stokes, Gregory Tierney, Ross Tucker, Ben Jones

**Affiliations:** 1 Carnegie Applied Rugby Research (CARR) Centre, Carnegie School of Sport, Leeds Beckett University, Leeds, UK; 2 Biomedical Engineering and Mechanics, Virginia Tech, Blacksburg, VA USA; 3 Leeds Rhinos Rugby League Club, Leeds, UK; 4 Sport and Exercise Sciences Research Institute, Ulster University, Belfast, UK; 5 Nanotechnology and Integrated Bioengineering Centre (NIBEC), School of Engineering, Ulster University, Belfast, UK; 6 School of Physical Education Sport and Exercise Sciences, University of Otago, Dunedin, New Zealand; 7 Centre for Health and Injury and Illness Prevention in Sport, University of Bath, Bath, UK; 8 World Rugby, 8-10 Pembroke St., Dublin, Ireland; 9 Sports Injury Prevention Research Centre, University of Calgary, Calgary, Canada; 10 Sydney School of Health Sciences, Faculty of Medicine and Health, The University of Sydney, Camperdown, NSW Australia; 11 Rugby Football League, Etihad Campus, Manchester, UK; 12 Medical Services, Rugby Football Union, Twickenham, UK; 13 Institute of Sport and Exercise Medicine, Department of Sport Science, University of Stellenbosch, Stellenbosch, South Africa; 14 Division of Physiological Sciences and Health through Physical Activity, Lifestyle and Sport Research Centre, Department of Human Biology, Faculty of Health Sciences, University of Cape Town, Cape Town, South Africa; 15 School of Behavioural and Health Sciences, Faculty of Health Sciences, Australian Catholic University, Brisbane, QLD Australia; 16 Premiership Rugby, London, UK

**Keywords:** Instrumented mouthguards, Video analysis, Brain injury, Head acceleration

## Abstract

**Purpose:**

Head acceleration events (HAEs) are a growing concern in contact sports, prompting two rugby governing bodies to mandate instrumented mouthguards (iMGs). This has resulted in an influx of data imposing financial and time constraints. This study presents two computational methods that leverage a dataset of video-coded match events: *cross-correlation synchronisation* aligns iMG data to a video recording, by providing playback timestamps for each HAE, enabling analysts to locate them in video footage; and *post-synchronisation event matching* identifies the coded match event (e.g. tackles and ball carries) from a video analysis dataset for each HAE, this process is important for calculating the probability of match events resulting in HAEs. Given the professional context of iMGs in rugby, utilising commercial sources of coded match event datasets may expedite iMG analysis.

**Methods:**

Accuracy and validity of the methods were assessed via video verification during 60 rugby matches. The accuracy of *cross-correlation synchronisation* was determined by calculating synchronisation error, whilst the validity of *post-synchronisation event matching* was evaluated using diagnostic accuracy measures (e.g. positive predictive value [PPV] and sensitivity).

**Results:**

*Cross-correlation synchronisation* yielded mean synchronisation errors of 0.61–0.71 s, with all matches synchronised within 3 s’ error. *Post-synchronisation event matching* achieved PPVs of 0.90–0.95 and sensitivity of 0.99–1.00 for identifying correct match events for SAEs.

**Conclusion:**

Both methods achieved high accuracy and validity with the data sources used in this study. Implementation depends on the availability of a dataset of video-coded match events; however, integrating commercially available video-coded datasets offers the potential to expedite iMG analysis, improve feedback timeliness, and augment research analysis.

**Supplementary Information:**

The online version contains supplementary material available at 10.1007/s10439-025-03679-1.

## Introduction

Head acceleration events (HAEs) are acceleration responses of the head caused by short-duration collision forces [1]. Particularly within contact sports, HAEs are a concern due to the potential long-term health consequences associated with them, such as chronic traumatic encephalopathy [2] and cognitive impairment [3].

Instrumented mouthguards (iMGs) have emerged as a tool for quantifying HAEs and their use has grown within research [4]. Furthermore, both World Rugby and Rugby Football League have made iMGs mandatory in their elite competitions [5, 6]. Each time an iMG detects acceleration exceeding a pre-determined trigger threshold, inertial sensors embedded within iMGs record a short period of kinematic data which is then written to fixed memory and stored as a sensor acceleration event (SAE). The magnitude and frequency of HAEs are then approximated using the dataset of recorded SAEs [7]. These data can subsequently inform policy decisions for mitigating HAE exposure.

**Fig. 1:**
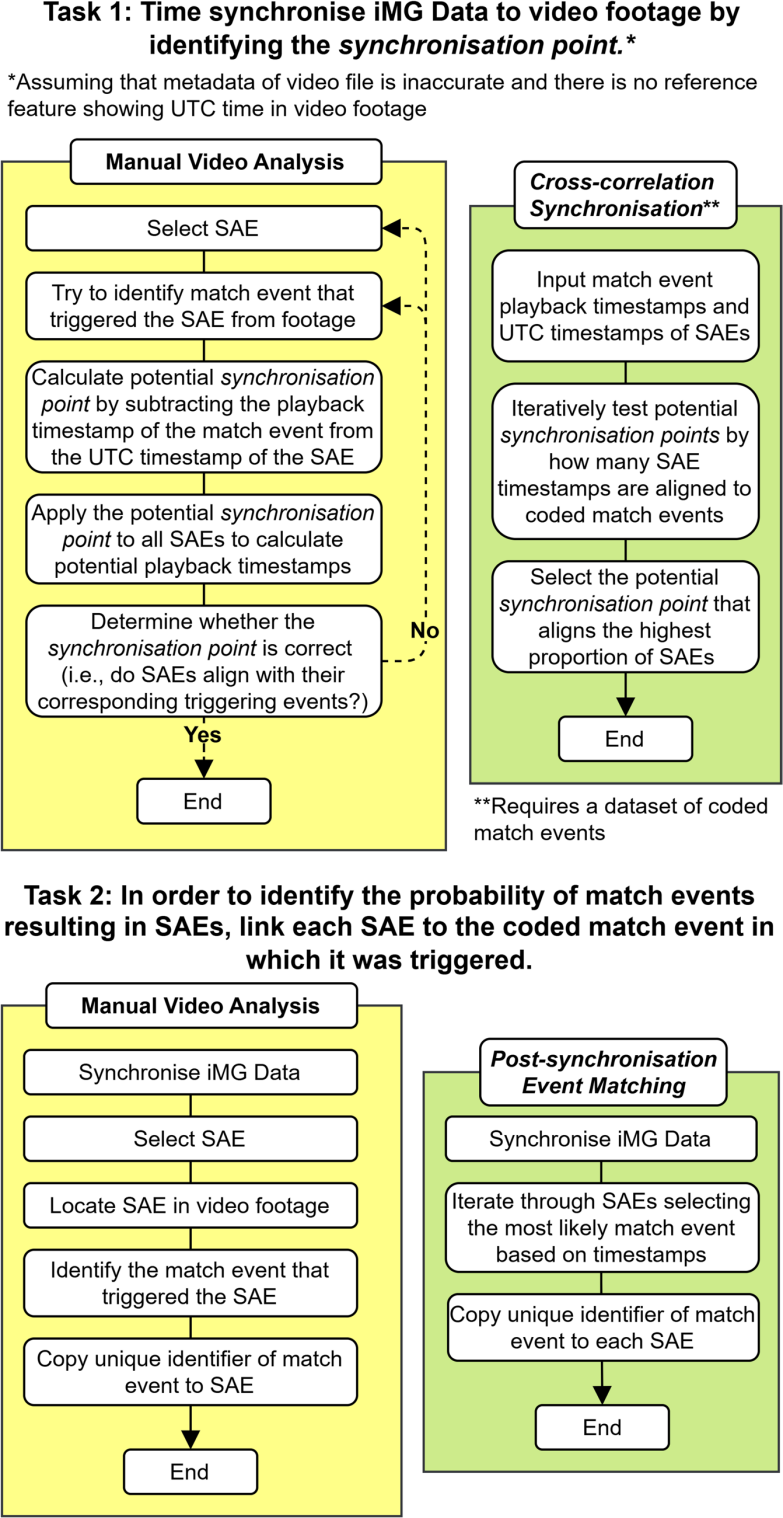
Manual video analysis approaches for two tasks involved in the analysis of iMG data with computational alternatives *cross-correlation synchronisation* and *post-synchronisation event matching*. These manual video analysis approaches have been used in the previous research [8–10], whereas the accuracy and validity of the computational approaches are the subject of this study. Note that the *synchronisation point* is the UTC value at the start of a video recording

Video analysis can be an important process for contextualising SAEs to enable a further understanding of HAE exposure in sports. For example, video analysis may be used to identify the match event during which each SAE was recorded (e.g. tackles and ball carries in rugby) [8, 9]. However, before video analysis of SAEs can take place, a synchronisation process is required to locate each SAE within the video recording. Synchronisation entails converting coordinated universal time (UTC) values provided in SAE timestamps (i.e. real-world time) to playback timestamps (i.e. time in video). This is achieved by subtracting the *synchronisation point* from the UTC timestamp of each SAE. The *synchronisation point* is the UTC value at the start of a video recording and may be recorded manually during data collection (e.g. filming a world clock at the start of the video), or retrospectively via metadata stored in the video file. However, both methods tend to be unreliable in professional settings as video is often recorded independently from iMG data collection (e.g. broadcast recording, provided by the teams involved, or provided by an organisation) and metadata may be altered during transfer and conversion. Consequently, the *synchronisation point* often needs to be ascertained retrospectively (i.e. Fig. 1, Task 1).

Following video analysis, statistical analysis to estimate the probability of match events (e.g. tackles and ball carries) resulting in HAEs can allow for higher-risk areas within sports to be identified. Probability has been calculated for match events during rugby union [11–13] matches and its calculation requires two processes: firstly, match events are coded from a video recording via video analysis; secondly, SAEs are linked to the video-coded match events during which the SAE was triggered (i.e. Fig. 1, Task 2). Subsequently, the probability of an HAE occurring during a match event is determined from the SAEs linked to each match event.

Synchronisation and video analysis required for calculating variables such as probability of HAE from specific events have typically been achieved using manual video analysis approaches in the previous research [8–10] (Fig. 1). These manual processes place significant demands on financial and time constraints for governing bodies and researchers. Recent iMG mandates [5, 6] have increased these demands further due to increased data collection. In these competitions, matches are often video analysed for commercial purposes, such as media coverage and sportsbooks. Due to the demand for these data, commercial providers perform video analysis of matches on a large scale with short deadlines. This process results in a dataset of video-coded match events, including a player identifier and playback timestamp for a variety of match events, such as ball carries and tackles. Leveraging datasets of coded match events to replace manual processes may expedite the analysis of iMG data, which is crucial in large-scale projects for informing policy decisions and improving the timeliness of feedback provided to players and medical staff.

In this study, two computational methods leveraging a commercial video analysis data source are provided as alternatives to manual processes (e.g. video analysis to identify match events for each SAE). Firstly, *cross-correlation synchronisation* is proposed as a computational alternative method to video synchronisation (Fig. 1, Task 1). This method can expedite the video analysis process by providing playback timestamps for SAEs, enabling analysts to locate SAEs within video footage. Secondly, *post-synchronisation event matching* is provided as a computational method for identifying the triggering event (i.e. the match event during which an SAE was recorded) of each SAE from a dataset of coded match events (Fig. 1, Task 2). This process is essential for calculating probability-based metrics to quantify the risk of match events resulting in HAEs. *Post-synchronisation event matching* requires SAE timestamps to be synchronised to the playback timestamps of coded match events and therefore can be used in conjunction with *cross-correlation synchronisation*. The implementation of both methods is contingent upon access to an appropriate dataset of video-coded match events. The aim of this study was to assess the accuracy of *cross-correlation synchronisation* and the validity of *post-synchronisation event matching* against their manual counterparts using iMG and commercial video analysis data collected from three rugby competitions.

## Methods

### Study Design

This study assessed the accuracy and validity of two computational processes, *cross-correlation synchronisation* and *post-synchronisation event matching*, by comparing their outputs with their manual counterparts. Analysis was conducted using iMG and video analysis data collected from 60 elite-level rugby matches during the 2023 season, including men’s rugby union (*n* = 20; Super Rugby, Australia, Fiji, New Zealand, and the Pacific Islands), women’s rugby union (n = 20; Farah Palmer Cup, New Zealand), and men’s rugby league (*n* = 20; Super League, England and France). Validation was conducted separately for rugby union and rugby league to determine the validity for each sport. No women’s rugby league video analysis data were available. For rugby union, men’s and women’s data were combined in the main manuscript and findings are provided separately in Supplementary Material.

All players in each club were offered iMGs as part of league-wide iMG programmes and player participation was voluntary, resulting in 300 participants (Super Rugby *n* = 72, Farah Palmer Cup *n* = 183, Super League *n* = 45) and 466 player matches (Super Rugby *n* = 151, Farah Palmer Cup *n* = 242, Super League *n* = 73). Participants provided written consent and ethics approval was received from the Leeds Beckett University Ethics Committee (REF #108638) and by World Rugby’s internal Ethics Committee.

### Instrumented Mouthguard Data

Prevent Biometrics (MN, USA) provided all iMGs and were fitted to players using 3D dental scans. The iMG devices were instrumented with accelerometers and gyroscopes, both sampling at 3200 Hz and with measurement ranges of ± 200 g and ± 35 rad/s, respectively. An SAE, composed of 50 ms of sensor data, was written to memory every time the accelerometer measures exceeded 8 g along any axis. In-house Prevent Biometric algorithms classified each SAE as a valid recording of head movement or non-head movement using proximity data captured by infrared sensors. Only SAEs classified as valid head movement by this algorithm were used in this study. Previous validations have shown high positive predictive values (PPV) with the current iMG system in both rugby union and rugby league, ranging from 91% [14] to 94% [10], which has been shown to increase to 99% by excluding SAEs that do not exceed 5 g and 400 rad/s^2^, as these SAEs are typically caused by non-contact [15]. Consequently, only SAEs recorded by the iMG which exceeded both 5 g (at the head centre of gravity) and 400 rad/s^2^ are used in this study. Sensitivity (i.e. the proportion of HAEs that were recorded as SAEs) has been shown to range from 75% [10] to 86% [8] using video analysis.

### Video Analysis

Video recordings (25 frames per second, with tight and wide angles) and coded match events were provided by Opta (StatsPerform, Chicago, IL). Opta data are collected for commercial purposes using video analysis and include coded events for a variety of match events across rugby union and rugby league competitions. Opta data has previously been validated in a previous study [16]. Henceforth, coded match events refer to Opta data. Playback timestamps and player identifiers were extracted from coded match events. For rugby league, match events included tackles and carries, and for rugby union, tackles, carries, and rucks were used.

**Fig. 2:**
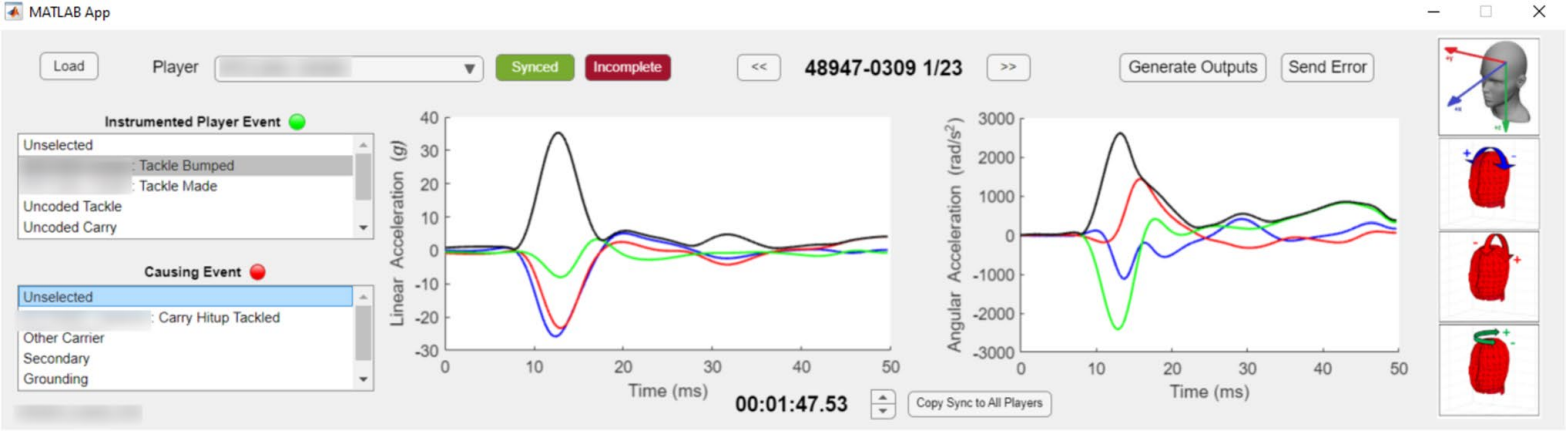
A bespoke MATLAB GUI used in conjunction with video recordings during manual synchronisation and labelling processes

A synchronisation process was required to align SAE timestamps (UTC) with timestamps of match events (playback timestamps), because UTC timestamps were not provided by Statsperform. All matches were synchronised using the manual video analysis method as shown in Fig. 1 (Task 1) and the MATLAB (R2023a version 9.14.0) graphical user interface (GUI) as shown in Fig. 2. Additionally, synchronisation was refined to a high degree of accuracy using frame-by-frame video playback alongside the GUI visualisation of directional data (Fig. 2), allowing the analyst to confirm that the head motion observed in video corresponded with the head kinematics from each SAE. This refinement was made separately for each device to account for variation between devices in UTC values. This process allowed analysts to identify the when SAEs were triggered within a tolerance of two frames (i.e. 0.08 s).

Video analysis was used to identify the triggering event for each SAE. This analysis was conducted by two experienced analysts using a GUI (Fig. 2). If the SAE was triggered during a coded match event, analysts labelled the SAE with the unique identifier specific to the coded match event. However, not every SAE was triggered by a coded match event, therefore these SAEs were labelled with the triggering event according to definitions provided in Table 1 (i.e. uncoded tackle, uncoded carry, uncoded ruck, aerial challenge, carry assist, lineout, maul, non-contact, off-the-ball, scrum). This analysis provided the criterion for assessing the validity of the *post-synchronisation event matching* method.

**Table 1:** The definitions of triggering events labelled during manual video analysis of SAEs

Triggering event	Definition
Coded tackle	The SAE was triggered by a tackle that was included within coded match events
Coded carry	The SAE was triggered by a carry that was included within coded match events
Coded ruck	The SAE was triggered by a ruck that was included within coded match events
Uncoded tackle	The SAE was triggered by a tackle was not included within coded match events
Uncoded carry	The SAE was triggered by a carry that was not included within coded match events
Uncoded ruck	The SAE was triggered by a ruck that was not included within coded match events
Aerial challenge (uncoded)	The SAE was triggered whilst the player was competing for the ball in the air (not during the lineout)
Carry assist (uncoded)	The SAE was triggered whilst the player was assisting a teammate carrying the ball before a ruck or maul had formed
Lineout (uncoded)	The SAE was triggered whilst the player was competing in a lineout
Maul (uncoded)	The SAE was triggered whilst the player was competing in a maul
Non-contact (uncoded)	The SAE was triggered by a ground reaction force through the players’ feet
Off-the-ball (uncoded)	The SAE was triggered by a collision away from the ball
Scrum (uncoded)	The SAE was triggered whilst the player was competing in a scrum

### Cross-correlation Synchronisation

**Fig. 3:**
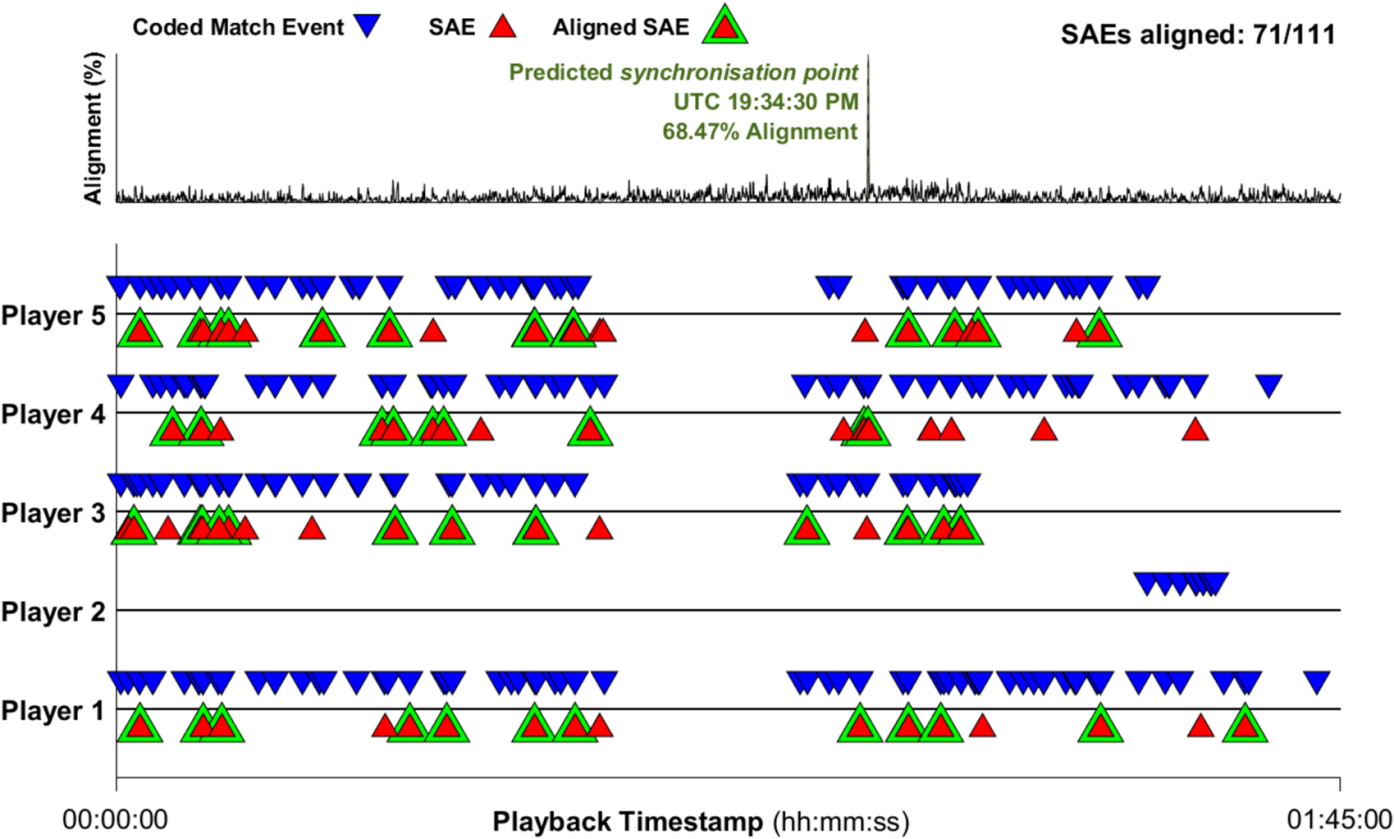
The predicted synchronisation point using the *cross-correlation synchronisation* method. Coded match events were extracted from coded match events. SAE—Sensor acceleration event, UTC—Universal Coordinated Time

*Cross-correlation synchronisation* is a computational solution that predicts a *synchronisation point* using UTC timestamps of SAEs and playback timestamps of coded match events via a cross-correlation process. This process entails iterating through potential synchronisation points to select the UTC value which aligns the most SAEs with coded match events for the same player. The UTC value with the highest proportion of aligned SAEs is selected as the predicted *synchronisation point* (Fig. 3).

*Cross-correlation synchronisation* was conducted for each match (*n* = 60) using the custom-built *synchronise* function (provided in Supplementary Material) within MATLAB. Given that tackle and carry events have been shown to have the highest probability of resulting in HAEs in both rugby union [8] and rugby league [9], player identifiers and playback timestamps of these events were used as inputs in the *synchronise* function.

### Post-synchronisation Event Matching

The *post-synchronisation event matching* method is a computational solution for identifying the triggering event of SAEs from video-coded match events. The triggering event is the match event during which the SAE was recorded (Table 1). The method predicts the triggering event of each SAE based on the timestamps of coded match events and SAEs. If the triggering event of an SAE was deemed to be a coded match event, the unique identifier of the coded match event is added as a label to the SAE to ‘link’ the events together, allowing for statistical analyses to estimate the probability of HAEs occurring from match events.

*Post-synchronisation event matching* was conducted using the custom-built *linkcollision* function (provided in Supplementary Material) within MATLAB. This function iterated through each SAE (*n* = 7248) and linked it to a coded match event if the timestamp of the SAE and the coded match event were within 7 s (before or after) of one another. If multiple match events occurred within this window, the coded match event with the closest playback timestamp was selected. The window of 7 s was selected based on preliminary analyses to optimise validity and may vary if different datasets are used. Playback timestamps of SAEs were obtained via *cross-correlation synchronisation* described in the previous section; therefore, any error was carried over from this process. The decision to use playback timestamps estimated via *cross-correlation synchronisation* instead of manually identified *synchronisation points* was made to test the validity of *post-synchronisation event matching* within an automated approach (i.e. using *cross-correlation synchronisation* alongside *post-synchronisation event matching*).

### Statistical Analysis

The accuracy of *cross-correlation synchronisation* was assessed by calculating synchronisation error for each match (*n* = 60, *n* SAEs per match ranged from 20 to 406). Synchronisation error was calculated as the absolute difference between *synchronisation points* obtained via *cross-correlation synchronisation* and manual video analysis-based synchronisation. Manual video-analysis-based synchronisation (Fig. 1) was conducted using the MATLAB GUI (Fig. 2) and involved frame-by-frame video analysis to synchronise each iMG device to video recordings within a tolerance of two frames (i.e. 0.08 s). This process revealed variation between *synchronisation points* of devices synchronised to the same video recording, with a standard deviation that ranged from 0.04 to 1.22 s across matches. Therefore, the mean *synchronisation point* across all devices for each match was used as the criterion value.

The validity of *post-synchronisation event matching* was assessed by comparing outputs with manual video analysis. This comparison resulted in the classification of each SAE as a true positive, false positive, true negative, or false negative, whose definitions are shown in Table 2. These classifications were used to assess the validity of *post-synchronisation event matching* when using manual video analysis as a criterion (Eqs. 1–5); they do not refer to the ‘ground truth.’ In the context of this study, PPV (Eq. 1) and sensitivity (Eq. 3) assessed the ability of *post-synchronisation event matching* to correctly identify SAEs that were triggered by coded match events, with PPV evaluating the correctness of these identifications and sensitivity evaluating the ability to detect these SAEs without missing any. Negative predictive value (NPV, Eq. 2) and specificity (Eq. 4) evaluated the ability to correctly identify SAEs that were not triggered by coded match events, with NPV evaluating the reliability of negative identifications and specificity evaluating the ability to accurately recognise true negatives. Accuracy (Eq. 5) evaluates the overall ability of *post-synchronisation event matching* to correctly identify both SAEs triggered by coded match events and those triggered by uncoded events, as compared to the video analysis labelling.

1 $$ {\text{PPV = }}\frac{{{\text{TP}}}}{{\text{TP + FP}}}, $$

2 $$ {\text{NPV = }}\frac{{{\text{TN}}}}{{\text{TN + FN}}}, $$

3 $$ {\text{Sensitivity}} = { }\frac{{{\text{TP}}}}{{\text{TP + FN}}}, $$

4 $$ {\text{Specificity}} = { }\frac{{{\text{TN}}}}{{\text{TN + FP}}}, $$

5 $$ {\text{Accuracy}} = { }\frac{{\text{TP + TN}}}{{\text{TP + FP + TP + FP}}}, $$

where $\text{TP}$ is the number of true positives, $\text{FP}$ is the number of false positives, $\text{TN}$ is the number of true negatives, and $\text{FN}$ is the number of false negatives.

**Table 2:** Classification of SAEs for assessing the validity of the *post-synchronisation event matching* method

Classification	Definition
True positive	*Post-synchronisation event matching* and video analysis identified the triggering event of an SAE to be the same coded match event
False positive	*Post-synchronisation event matching* identified the triggering event of an SAE to be a coded match event, whereas video analysis identified the triggering event to be either a different coded match event or an uncoded event
True negative	*Post-synchronisation event matching* did not identify a coded match event as the triggering event, and video analysis identified the triggering event to be an uncoded event
False negative	*Post-synchronisation event matching* did not identify a coded match event as the triggering event, but video analysis did

## Results

**Fig. 4:**
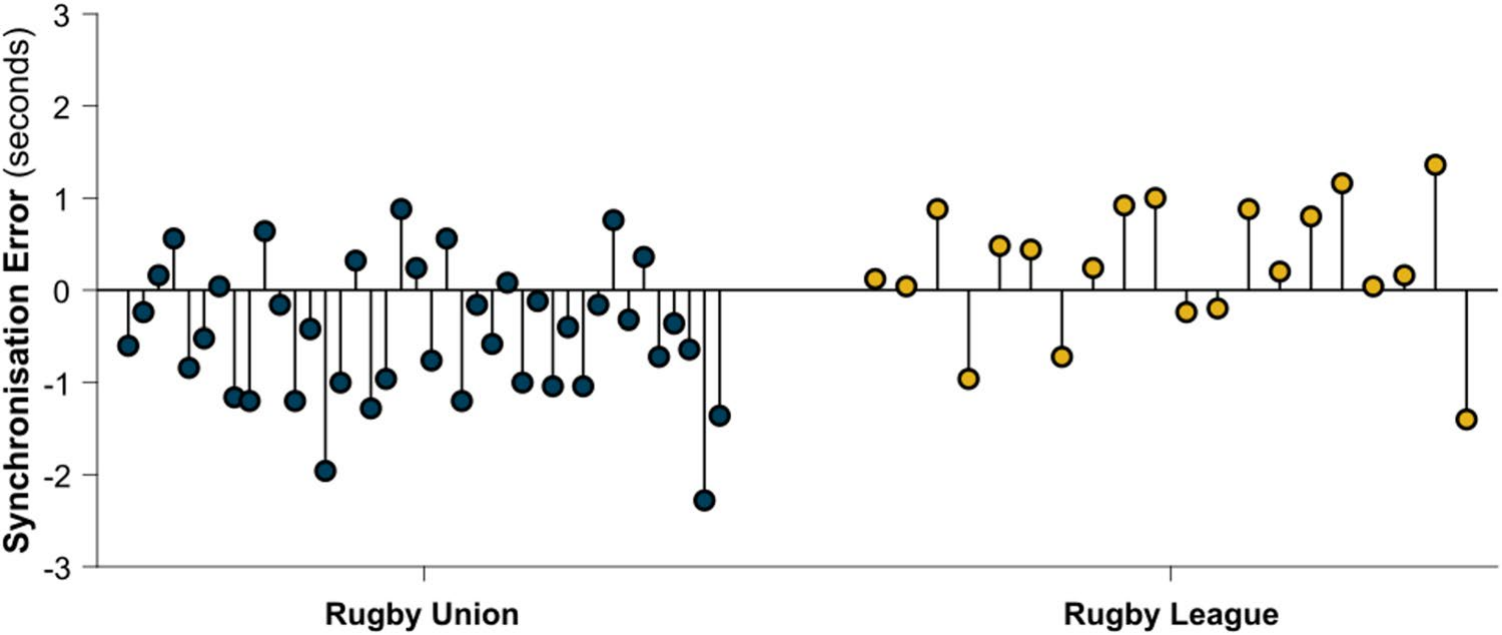
Synchronisation error for each match following *cross-correlation synchronisation* in rugby union (blue) and rugby league (yellow).

The mean (± standard deviation) synchronisation error of *cross-correlation synchronisation* was 0.71 s (± 0.50) in rugby union matches and 0.61 s (± 0.45) in rugby league matches. Across all matches, 78.3% (*n* = 47) were synchronised within a 1-s error and 98.3% (*n* = 59) were synchronised within a 2-s error, with one match having a synchronisation error of 2.3 s (Fig. 4).

The validity of *post-synchronisation event matching* is shown in Table 3. The overall accuracy was 0.92 and 0.95 in rugby union and rugby league, respectively. In rugby union, PPV was 0.90 compared to 0.95 in rugby league. The slightly lower PPV is due to a higher proportion of false positives (i.e. SAEs linked to the incorrect coded match event) in rugby union than in rugby league. Conversely, there were a higher proportion of true positives and a lower proportion of true negatives in rugby league compared to rugby union.

**Table 3:** The validity of *post-synchronisation event matching* compared to a manual video analysis-based approach

	Rugby union	Rugby league
True positive	67.88% (*n* = 3780)	84.63% (*n* = 1421)
False positive	7.70% (*n* = 429)	4.17% (*n* = 70)
True negative	24.19% (*n* = 1347)	10.54% (*n* = 177)
False negative	0.23% (*n* = 13)	0.66% (*n* = 11)
PPV	0.90	0.95
NPV	0.99	0.94
Sensitivity	1.00	0.99
Specificity	0.76	0.72
Accuracy	0.92	0.95

**Fig. 5:**
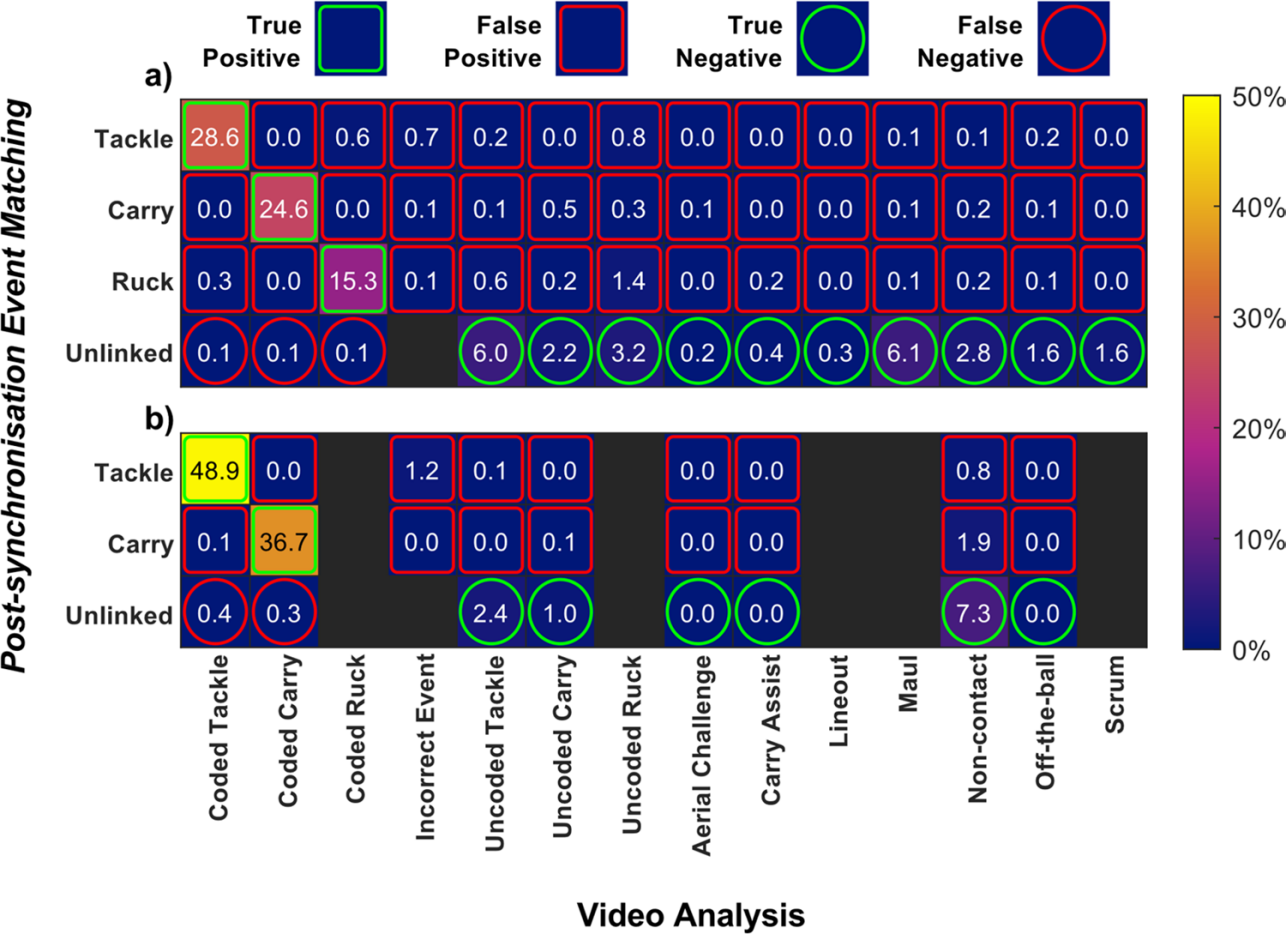
Confusion matrices with a heatmap showing the proportion of SAEs classified by the triggering event using the *post-synchronisation event matching* method (rows) and manual video analysis (columns) for rugby union (**a**) and rugby league (**b**). Classifications used for validity are shown by overlaid circles and squares. Triggering event definitions are provided in Table 1 and classification definitions are shown in Table 2. The incorrect event column included SAEs that had the same match event type (i.e. coded tackles, carries, and rucks), but were linked to a different video-coded event by each method.

Figure 5 is a confusion matrix heatmap comparing the classification of SAEs via the *post-synchronisation event matching* method (row values) against manual video analysis (column values). For example, the first cell with the value of 28.6% illustrates that 28.6% of SAEs in rugby union were classified as coded tackles by *post-synchronisation event matching* and also by manual video analysis; the cell two to the right of it, 0.6%, shows that 0.6% of SAEs in rugby union were classified as a coded tackle by *post-synchronisation event matching* and as a coded ruck by manual video analysis.

Across men’s and women’s rugby union, 70.08% (*n* = 3780) SAEs were triggered by a coded match event, compared to 86.60% (*n* = 1454) in men’s super league. Non-contact events accounted for 9.89% (*n* = 166) of SAEs in rugby league and 3.34% (*n* = 186) of SAEs across rugby union. Match events other than those included within coded match events such as aerial challenges, carry assists, lineouts, mauls, off-the-ball collisions, and scrums accounted for 14.56% (*n* = 811) of SAEs across rugby union and none in rugby league. Tackles, carries, and rucks that were not included as coded match events (i.e. uncoded tackles, carries, and rucks) accounted for 3.51% (*n* = 59) of SAEs in men’s rugby league and 15.35% (*n* = 855) of SAEs across rugby union.

## Discussion

This study assessed the accuracy and validity of two computational methods leveraging a dataset of coded match events. The mean error associated with *cross-correlation synchronisation* was 0.71 s in rugby union and 0.61 s rugby league, with 98.3% of matches being synchronised within a 2-s error and all matches being synchronised within 3 s. The validity of *post-synchronisation event matching* was assessed by comparing outputs against manual video analysis, resulting in accuracy values of 0.92 in rugby union and 0.95 in rugby league. Overall, this study demonstrated that computational processes leveraging commercial video analysis data (*cross-correlation synchronisation* and *post-synchronisation event matching*) have high agreement with their manual video analysis counterparts. These methods are dependent on the availability of a dataset of video-coded match events, however, given the context of mandated iMGs in professional rugby [5, 6], these datasets may be available commercially. Implementing *cross-correlation synchronisation* and *post-synchronisation event matching* can replace manual processes and expedite the analysis of iMG data from professional rugby union and rugby league competitions, improving the timeliness of feedback provided to players and medical staff and for informing policy decisions and augment processes for research using iMGs. This is pertinent given the recent mandates of iMGs within these sports [5, 6].

The accuracy of *cross-correlation synchronisation* was similar across rugby union and rugby league, with mean synchronisation errors of 0.72 and 0.61 s, respectively. All matches in the study were synchronised to within 3-s error, indicating good reliability of predicted *synchronisation points*. If *cross-correlation synchronisation* is used solely to prepare SAEs for video analysis, then synchronisation error within 3 s should be adequate for locating SAEs within a video recording, especially if directional information from time series data are used alongside video analysis (Fig. 2). Another application of *cross-correlation synchronisation* can be to align SAEs that have inaccurate UTC timestamps. For example, there is a known issue with Prevent Biometrics iMGs that causes incorrect UTC timestamps: if the device operates without connecting to an iOS device during a battery cycle, it defaults to recording SAEs with timestamps starting from the Unix epoch (January 1, 1970). If the correct date for SAEs can be identified, *cross-correlation synchronisation* can be used to realign UTC timestamps using known UTC timestamps of video-coded match events.

Despite the maximum synchronisation error being less than 3 s, *cross-correlation synchronisation* was unable to consistently achieve a synchronisation error less than 1 s. This was likely due to a lack of precision between the timestamps of coded match events and when the SAE was triggered. For example, video analysts may not code the match event the exact moment it occurs, additionally, an SAE may be triggered during the grounding stage of a tackle or ball carry [8, 9], whereas the playback timestamp of the tackle or ball carry may relate to the moment of initial collision. If more accurate *synchronisation points* are required, then manual adjustments made using frame-by-frame video analysis and directional data can achieve this. These adjustments would need to be made separately for each device to account for variations between devices in UTC values.

The validity of *post-synchronisation event matching* can be assessed using PPV (0.90 and 0.95), NPV (0.99 and 0.94), sensitivity (1.00 and 0.99), specificity (0.76 and 0.72), and accuracy (0.92 and 0.95) values provided; however, whether values are deemed acceptable may vary depending on the research question or application. One application for *post-synchronisation event matching* may be to understand the probability of match events resulting in SAEs exceeding a given magnitude [8, 11]. For this application, the ability of *post-synchronisation event matching* to correctly identify SAEs that were triggered by coded match events is important and can be accessed via sensitivity and PPVs. Sensitivity values of 1.00 (rugby union) and 0.99 (rugby league) indicate that the method was effective at detecting all SAEs that were triggered by coded match events across both rugby union and rugby league, whilst PPVs of 0.90 and 0.95 indicate that most SAEs were linked to the correct coded match event. Slightly lower PPVs than sensitivity values reflect a tendency to record false positives (i.e. when SAEs are linked to the incorrect coded match event). When estimating the probability of match events resulting in HAEs, the presence of these false positives may cause probabilities to be overestimated. However, given that known iMG limitations, such as the linear acceleration trigger bias [17] and the rearming period [7], contribute to the underestimation of HAEs, overestimation may be deemed more acceptable than further underestimation. Lower specificity values (0.72 and 0.76) are less of a concern as they evaluate the ability of *post-synchronisation event matching* to identify true-negative cases (i.e. SAEs that were not linked to a coded match event by *post-synchronisation event matching* or manual video analysis).

Another application of *post-synchronisation event matching* may be to understand the context surrounding SAEs. However, for this purpose *post-synchronisation event matching* is constrained by the dataset of coded match events. A major issue for this application is that not every triggering event was included within the coded match event data provided by Opta. This meant that not every SAE could be characterised using the *post-synchronisation event matching* method. This was less of an issue in men’s rugby league, where 86.60% of SAEs were triggered by coded match events, compared to 70.08% across rugby union SAEs. In rugby league, 9.89% of all SAEs were caused by non-contact events (e.g. running, jumping, or changing direction), these SAEs are typically low in magnitude and removed using thresholds [18]. However, the fact that some SAEs triggered by non-contact events remained in this dataset despite the use of a 5 g and 400 rad/s^2^ threshold aimed to remove them may suggest a higher threshold is needed. In rugby union, 14.56% of all SAEs were triggered by events outside of the coded match event, such as aerial challenges, carry assists, lineouts, mauls, off-the-ball collisions, and scrums. These events represent a limitation of the current video analysis dataset. Further video analysis would be required to identify the triggering event for these SAEs. Similarly, for rugby union and rugby league, respectively, 15.35 and 3.51% of all SAEs were triggered by match events that are usually coded but were not present in the coded match event dataset. It is unclear why some tackles, carries, and rucks were not included within the coded match event dataset, however, speculatively, these may be due to events being coded with the wrong player identifier, missed entirely by the video analyst, or due to the events occurring outside of normal play (i.e. events occurring during advantage periods following a foul that is subsequently given are not included within the coded match event dataset used in this study). These missing match events reduce the number of SAEs that can be characterised via *post-synchronisation event matching* and may mean that the sample of match events included within coded match events may not be entirely representative of all match events during a match.

## Limitations

This study demonstrates that two automated processes can be effective for replacing current manual processes if an appropriate dataset of coded match events is available. However, these findings are specific to the iMG system, coded match event provider, and rugby code used in this study. If these processes are to be implemented using different sources of data (i.e. iMG system or coded match event provider), separate validation would be required, and the current methodology may serve as a protocol.

Whether the methods can be effective may be influenced by several factors. For example, in this study, the iMG manufacturer’s algorithm to determine whether SAEs were true positives (i.e. triggered during HAEs) was effective for rugby union and rugby league [10, 14], whereas other SAE datasets (i.e. different sports or iMG systems) may contain more false positives. In the context of this study, false-positive SAEs would not have a corresponding coded match event. However, findings from this study may suggest that this may not necessarily impair performance of *cross-correlation synchronisation*. The datasets in this study contained a high proportion of SAEs not having a corresponding coded match event (i.e. 48.68% in rugby union); however, despite this, high accuracy was still achieved by *cross-correlation synchronisation*. This is because the probability of multiple SAEs erroneously being aligned to coded match events is low. Consequently, the most important factor for achieving a low synchronisation error is to have multiple SAEs which do have corresponding coded match events. This may be more difficult to achieve in sports with fewer HAEs. The lowest HAE count for this study was 20 in a match.

Secondly, whilst this study demonstrated good agreement between the computational process and manual video analysis, it also revealed limitations of the external video analysis dataset. Specifically, there were a high number of SAEs that were caused by events that were not included within the coded match event dataset, particularly within rugby union. Further video analysis would be required to characterise these SAEs; however, *cross-correlation synchronisation* and *post-synchronisation event matching* can still expedite this process. Finally, this study assessed the accuracy and validity of computational processes by comparing them with manual video analysis processes. Whilst video analysis has been used in previous research for these processes, they may contain errors themselves due to the limitations of video analysis [19].

## Conclusion

This study demonstrated that two computational processes can be effective for replacing previously used manual processes if a dataset of coded match events are available. The mean error associated with *cross-correlation synchronisation* was 0.61 to 0.71 s and all matches were synchronised within 3 s. The validity of *post-synchronisation event matching* resulted in PPVs ranging from 0.90 to 0.95, NPVs ranging from 0.94 to 0.99, specificity values ranging from 0.72 to 0.76, sensitivity values ranging from 0.99 to 1.00, and accuracy values ranging from 0.92 to 0.95. High sensitivity and PPVs indicate that this method can be effective for identifying the coded match event for each SAE, allowing for the estimation of the probability of coded match events resulting in SAEs. However, further video analysis would be required to characterise SAEs that were triggered by events not included within the coded match event dataset. *Post-synchronisation event matching* used timestamps obtained via *cross-correlation synchronisation*. Therefore, if both processes are implemented simultaneously alongside commercial video analysis data, the analysis of iMG data can be automated to improve the timeliness and scalability of feedback provided to players and medical staff and for informing policy decisions.

## Supplementary Information

Below is the link to the electronic supplementary material.

Supplementary file1 (PDF 365 KB)

## Data Availability

The data that support the findings of this study are available from the corresponding author, JT, upon reasonable request.
