# Correction: Optimising Instrumented Mouthguard Data Analysis: Video Synchronisation Using a Cross-correlation Approach

**DOI:** 10.1007/s10439-025-03690-6

**Published:** 2025-01-30

**Authors:** James Tooby, Steve Rowson, Kevin Till, David Allan, Melanie Dawn Bussey, Dario Cazzola, Éanna Falvey, Kenzie Friesen, Andrew J. Gardner, Cameron Owen, Gregory Roe, Thomas Sawczuk, Lindsay Starling, Keith Stokes, Gregory Tierney, Ross Tucker, Ben Jones

**Affiliations:** 1 Carnegie Applied Rugby Research (CARR) Centre, Carnegie School of Sport, Leeds Beckett University, Leeds, UK; 2 Biomedical Engineering and Mechanics, Virginia Tech, Blacksburg, VA USA; 3 Leeds Rhinos Rugby League Club, Leeds, UK; 4 Sport and Exercise Sciences Research Institute, Ulster University, Belfast, UK; 5 Nanotechnology and Integrated Bioengineering Centre (NIBEC), School of Engineering, Ulster University, Belfast, UK; 6 School of Physical Education Sport and Exercise Sciences, University of Otago, Dunedin, New Zealand; 7 Centre for Health and Injury and Illness Prevention in Sport, University of Bath, Bath, UK; 8 World Rugby, 8-10 Pembroke St., Dublin, Ireland; 9 Sports Injury Prevention Research Centre, University of Calgary, Calgary, Canada; 10 Sydney School of Health Sciences, Faculty of Medicine and Health, The University of Sydney, Camperdown, NSW Australia; 11 Rugby Football League, Etihad Campus, Manchester, UK; 12 Medical Services, Rugby Football Union, Twickenham, UK; 13 Institute of Sport and Exercise Medicine, Department of Sport Science, University of Stellenbosch, Stellenbosch, South Africa; 14 Division of Physiological Sciences and Health through Physical Activity, Lifestyle and Sport Research Centre, Department of Human Biology, Faculty of Health Sciences, University of Cape Town, Cape Town, South Africa; 15 School of Behavioural and Health Sciences, Faculty of Health Sciences, Australian Catholic University, Brisbane, QLD Australia; 16 Premiership Rugby, London, UK

**Correction to: Annals of Biomedical Engineering** 10.1007/s10439-025-03679-1

The original article has been updated to add the missing Electronic Supplemental Material.

