# Supplementary material for: Optimising Instrumented Mouthguard Data Analysis: Video Synchronisation Using a Cross-correlation Approach
Source: Ann Biomed Eng. 2025 Jan 21;53(4):923–33. doi: 10.1007/s10439-025-03679-1 (PMC11929623; doi:10.1007/s10439-025-03679-1)
Supplement: Supplementary file 1 — Supplementary file1 (PDF 365 KB) [file 10439_2025_3679_MOESM1_ESM.pdf]

Optimising Instrumented Mouthguard Data Analysis: Video Synchronisation Using a Cross Correlation Approach

Supplementary Material

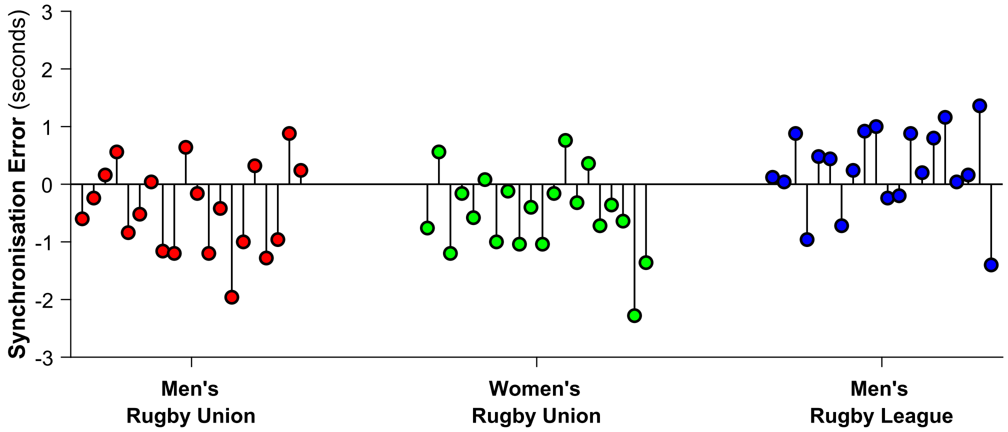

Supplementary Figure 1. Synchronisation error following *cross-correlation synchronisation* in men's rugby union (red), women's rugby union (green), and men's rugby league (blue) matches.

Supplementary Table 1. The validity of *post-synchronisation event matching* compared to a manual video-analysis based approach.

	**Men's Rugby Union**	**Women's Rugby Union**	**Men's Rugby League**
True Positive	66.87% (*n* = 2,080)	66.69% (*n* = 1,700)	84.63% (n = 1,421)
False Positive	7.65% (*n* = 231)	7.77% (*n* = 198)	4.17% (n = 70)
True Negative	23.21% (*n* = 701)	25.34% (*n* = 646)	10.54% (n = 1,77)
False Negative	0.26% (*n* = 8)	0.20% (*n* = 5)	0.66% (n = 11)
PPV	0.90	0.90	0.95
NPV	0.99	0.99	0.94
Sensitivity	1.00	1.00	0.99
Specificity	0.75	0.77	0.72
Accuracy	0.92	0.92	0.95

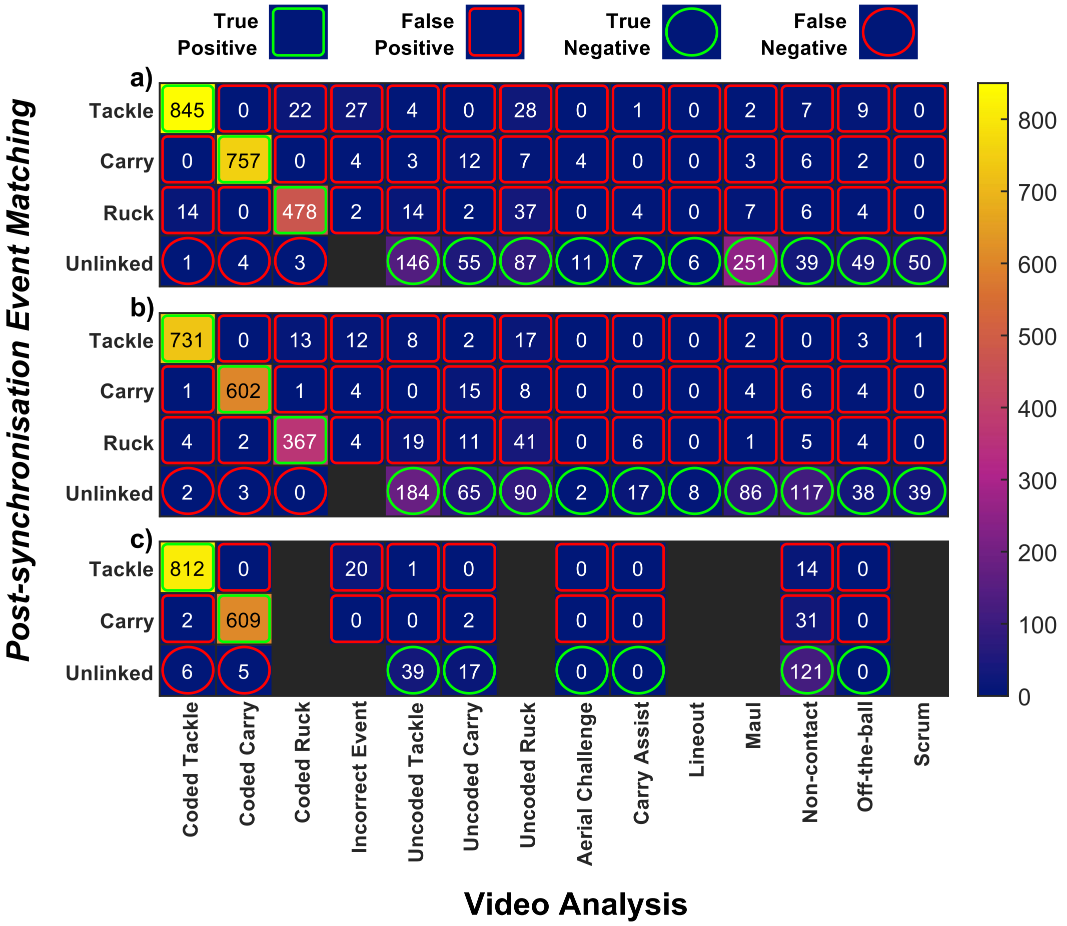

Supplementary Figure 2. Confusion matrices with a heatmap showing the identification of the triggering event across all SAEs via the *post-synchronisation event matching* method (rows) and manual video analysis (columns) for men's rugby union (a), women's rugby union (b), and men's rugby league (c). Classifications used for validity are shown by overlaid circles and squares. Triggering event definitions are provided in Table 1 and classification definitions are shown in Table 2. The incorrect event column included SAEs that had the same type of collision type (i.e. coded tackles, carries, and rucks), but were linked to different video-coded event by each method.

The *synchronise* function used to predict synchronisation points using *cross-correlation synchronisation*

```
function [predicted_syncpoint,max_alignment_prctage] = ...
        synchronise(collisions,saes,collisions_identifier,sae_identifier)

            %%%% Input arguments:

            % collisions
                  % playback timestamp of all collision events (in seconds)
            % saes
                  % UTC timestamp of all collision events (in seconds)
            % collisions_identifier
                  % player identifier for each collision event
            % sae_identifier
                  % player identifier for each SAE

            %%%% Output arguments:

            % predicted_syncpoint
                  % Predicted UTC timestamp at the start of the video

            % max_alignment_prctage (used for sanity checking prediction)
                  % Percentage of saes that are aligned to collision

            % magic numbers:
            alignment_window = seconds(duration(0,0,2));
                  % window to determine if SAE is aligned to collision start_point =
            seconds(duration(0,0,0));
                  % first potential sync point
            end_point = seconds(duration(24,0,0));
                  % last potential sync point intervals
            = seconds(duration(0,0,1));

                  % time between each sync point

            % pre-allocate vector of potential sync points potential_sync_points =
            linspace(start_point,end_point,...
                                                    (end_point - start_point)/intervals + 1)
            alignment_prctage = zeros([1 length(potential_sync_points)]); for i =
            1:length(potential_sync_points)

                  % apply potential sync
                  sae_playback = saes - potential_sync_points(i);
                        % playback timestamp of saes
                  % pre-allocate alignment check aligned_saes =
                  false([length(saes) 1]);

                  % alignment check
                        % > 0 collision events within alignment_window and with
                        % same identifier
                  for j = 1:length(sae_playback)
                        id = sae_identifier(j) == collisions_identifier & ...
                               abs(sae_playback(j) - collisions) < alignment_windo
                        aligned_saes(j) = sum(id) > 0;
          end

          alignment_prctage(i) = sum(aligned_saes)/length(sae_playback
     end
end
```

The *linkcollision* function used to predict the triggering event using the *post-synchronisation event matching* method

```
function predicted_collision_id = linkcollision(...
      collisions_id,collisions_timestamp,saes,collisions_player,sae_player

            %%%% Input arguments:

            % collisions_id
                  % unique identifer for each collision event
            % collisions_timestamp
                  % playback timestamp of all collision events (in seconds)
            % saes
                  % playback timestamp of all collision events (in seconds)
            % collisions_player
                  % player identifier for each collision event
            % sae_player
                  % player identifier for each SAE

            %%%% Output arguments:

            % predicted_collision_id
                  % identifier of the collision event predicted to have trigge
                  % each sae

            % magic numbers:
                  lead_time = seconds(duration(0,0,7)); lag_time =
                  seconds(duration(0,0,7));
                  % time in which collision events are considered to have ...
                  % triggered the sae:

            predicted_collision_id = zeros(length(saes),1); for i = 1:length(saes)

                  % for each sae sync and predict the collision event

                  % get collisions within lead and lag time
                  id = collisions_player == sae_player(i) & ... collisions_timestamp > saes - lead_time & ...
                         collisions_timestamp < saes + lag_time;

                  potential_collisions_timestamp = collisions_timestamp(id); potential_collisions_id =
                  collisions_id(id);

                  % if there are no collisions within lead and lag times
                  if isempty(potential_collisions_timestamp)
                        predicted_collision_id(i) = NaN; continue
                  end

                  % if there are multiple then choose the closest if
                  length(potential_collisions_timestamp) > 1
                        [~,id] = ... min(abs(seconds(potential_collisions_timestamp)-saes(i))
                        potential_collisions_id = collisions_id(id);
                  end

                  predicted_collision_id(i) = potential_collisions_id;

            end
end
```
